# In-Situ Nano-Auger Probe of Chloride-Ions during CH_3_NH_3_PbI_3−x_Cl_x_ Perovskite Formation

**DOI:** 10.3390/ma14051102

**Published:** 2021-02-26

**Authors:** Devthade Vidyasagar, Yong-Han Yun, Seunghak Shin, Jina Jung, Woosung Park, Jin-Wook Lee, Gill Sang Han, Changhyun Ko, Sangwook Lee

**Affiliations:** 1 School of Materials Science and Engineering, Kyungpook National University, Daeug 41566, Korea; vidyasagar.devtade@gmail.com (D.V.); 2007037233@knu.ac.kr (Y.-H.Y.); seunghak@knu.ac.kr (S.S.); joan219@knu.ac.kr (J.J.); 2 Division of Mechanical Systems Engineering, Sookmyung Women’s University, Seoul 04310, Korea; wpark@sookmyung.ac.kr; 3 Institute of Advanced Materials and Systems, Sookmyung Women’s University, Seoul 04310, Korea; 4 SKKU Advanced Institute of Nanotechnology (SAINT) and Department of Nanoengineering, Sungkyunkwan University, Suwon 16419, Korea; jw.lee@skku.edu; 5 School of Advanced Materials Science & Engineering, Sungkyunkwan University, Suwon 440-746, Korea; 6 Department of Applied Physics, College of Engineering, Sookmyung Women’s University, Seoul 04310, Korea

**Keywords:** perovskite, MAPbI_3−x_Cl_x_, nano-auger

## Abstract

Organo-halide perovskite solar cells (PSCs) have emerged as next-generation photovoltaics, owing to their high power-conversion efficiency (PCE), lower production cost, and high flexibility. ABX_3_-structured methylammonium lead triiodide (CH_3_NH_3_PbI_3_ or MAPbI_3_) perovskite is a widely studied light-absorbing material in PSCs. Interestingly, a small amount of chlorine incorporation into MAPbI_3_ increases charge carrier diffusion lengths (from 129 nm to 1069 nm), which enables planar structured PSCs with high PCEs. However, existence of chloride ions in the final perovskite film is still under debate. Contrastingly, few studies reported a negligible amount or absence of chloride ions in the final film, while others reported detection of chloride ions in the final film. Herein, we observed the microstructure and chlorine content of MAPbI_3−x_Cl_x_ thin films with increasing temperature via an in-situ nano-Auger spectroscopy and in-situ scanning electron microscopic analysis. The relative precipitation of MAPbI_3−x_Cl_x_ films occur at lower temperature and MAPbI_3−x_Cl_x_ grains grow faster than those of MAPbI_3_ grains. Local concentrations of chlorine at intragrain and the vicinity of grain boundary were analyzed to understand the behavior and role of the chloride ions during the microstructural evolution of the MAPbI_3−x_Cl_x_ films.

## 1. Introduction

Organo-halide perovskite (OHP) materials are a hot topic in photovoltaic research due to their excellent light harvesting ability and relatively lower processing costs [1,2,3]. In the past few years, perovskite solar cells (PSCs) have seen tremendous uplift in certified power conversion efficiencies (PCE), less than 14% in 2013 to >25% in 2020 [4,5,6]. The archetypal ABX_3_-structured methylammonium lead halides such as MAPbI_3_ and MAPbI_3−x_Cl_x_ are the most intensely studied reference materials in PSCs, owing to their remarkable optoelectronic properties [7,8]. MAPbI_3_ has large light-absorption coefficients (~10^5^ cm^−1^ at 550nm) due to a direct bandgap (~1.55 eV) between the valence (Pb s and I p orbital) and the conduction (Pb p orbital) band edge where intramolecular charge transfer occurs between the high-density orbitals [9,10]. Importantly, carrier diffusion length and band gap of MAPbI_3_ are controlled by compositional engineering. The fine control over optoelectronic properties and ease of synthesis has driven MAPbI_3_ to broad applications, including PSCs [11], solar-water splitting [12], lasing [13], and light-emitting diodes [14]. In addition, the incorporation of a small amount of bromine or chlorine into MAPbI_3_ is known to improve device properties [15]. Since the first report on PSCs using mixed halide MAPb_3−x_Cl_x_ as a light absorber, the PCE of the MAPb_3−x_Cl_x_ devices has already exceeded 18% [16,17]. The mixed halide MAPbI_3−x_Cl_x_ was found to have long carrier diffusion length of 1µm, about one order of magnitude longer than pure MAPbI_3_ system and 2.5 times higher diffusion constants (from 0.017 cm^2^s^−1^ to 0.042 cm^2^s^−1^) compared to pure MAPbI_3_ [18]. Despite many investigations, the role of chloride ions in MAPbI_3−x_Cl_3_ mixed-halide perovskites has not been rationalized [19,20,21,22]. Understandably, the ambiguity about chloride ions in MAPbI_3−x_Cl_x_ films is due to inefficient analytical techniques. As such, the concentration of chloride ions in MAPbI_3−x_Cl_x_ films after the annealing process is below the detection limit of an energy dispersive spectrometer (EDS) and X-ray photoelectron spectroscopy (XPS). Hence, to examine the role and volatilization behavior of chloride ions, it is desirable to have in-situ measurement techniques that are better than the conventional scanning electron microscopy (SEM) or EDS.

Herein, we use in-situ SEM and high-resolution in-situ nano-Auger electron spectroscopy (n-AES) to explore the role of chloride ions during MAPbI_3−x_Cl_x_ thin film formation. The surface microstructures were analyzed by in-situ SEM, quantitative components were analyzed using in-situ n-AES, and the structural analysis was performed using the X-ray diffraction (XRD) technique. The microstructural analysis was carried out by increasing the annealing temperature in-situ to confirm the volatilization behavior of chloride ions with increasing temperature. To quantitate chloride ions in MAPbI_3−x_Cl_x_ thin films, samples were in-situ annealed at a constant temperature over a period of time. During the volatilization, a nano-sized, high-resolution in-situ nano-auger system was used to quantitatively analyze the local concentration of chloride ions between the grain interior and grain boundary. The microstructure, grain growth, and diffusion or sublimation behavior of chloride ions during MAPbI_3−x_Cl_x_ thin film formation are succinctly discussed.

## 2. Materials and Methods

### 2.1. Materials

Methylammonium iodide (MAI, ≥99.5%) was purchased from Xi’an Polymer Light Technology Corp., Xi’an, China. Lead iodide (PbI_2_, 99.9985%) and lead chloride (PbCl_2_, 98%) were purchased from Alfa-Aesar, Ward Hill, MA, USA. Titanium (IV) diisopropoxide bis (acetylacetonate) (75 wt.%) and 1-butanol (99.8%) for the TiO_2_ blocking layer were purchased from Sigma-Aldrich, St. Louis, MO, USA. N, N-Dimethylformamide (DMF, 99.8% anhydrous), dimethyl sulfoxide (DMSO, ≥99.9% anhydrous), and diethyl ether (DEE, ≥99.7 anhydrous) were purchased from Sigma-Aldrich, St. Louis, MO, USA.

### 2.2. Preparation of TiO_2_ Blocking Layer

All the samples were prepared on TiO_2_ blocking layer-coated Silicon (n-type, resistivity = 0.007 ~ 0.002 Ω·cm, LG Siltron) substrates. The TiO_2_ blocking layer was prepared via the spin-coating of a 0.15 M titanium (IV) diisopropoxide bis(acetylacetonate) (75 wt.%) in 1-butanol (Sigma-Aldrich, 99.8%) on Si substrates. The TiO_2_ blocking layer precursor solution was spin-coated at 3000 rpm for 20s followed by soft baking at 125 °C for 5 min and hard baking at 500 °C for 30 min.

### 2.3. Formation of MAPbI_3−x_Cl_x_ Layer

The MAPbI_3−x_Cl_x_ precursor solution was prepared with 1:1:1 molar ratio of MAI, PbI_2_+PbCl_2_, and DMSO in DMF solvent at room temperature. The chlorine was added at 0.01 at% and 0.10 at%. Here, at% means atomic percentage of “X” cite in the perovskite. The MAPbI_3−x_Cl_x_ precursor solution was spin-coated at 4000 rpm for 20 s. To correlate our nano-AES analysis with the latest developments, we used an antisolvent method to fabricate a high-quality perovskite layer. During the spin coating, 500 µL of DEE was dropped on to the MAPbI_3−x_Cl_x_ solution. After the spin-coating, all the samples were soft-baked at 65 °C for 1 min. For the in-situ study, soft-baked samples were installed in an in-situ system. For other analyses, soft-baked samples were hard-baked at 130 °C for 10 min under vacuum (<10^−3^ Torr).

### 2.4. Characterization

The structural properties were measured by XRD (X’pert, Pan analytical, Amelo, The Netherlands). The microstructural and the thickness of the thin films were measured using filed-emission scanning electron microscopy (FE-SEM; JEOL, JSM-6701F, Tokyo, Japan). In-situ microstructural imaging was conducted by conventional SEM (Zeiss, Gemini Supra 55 VP-SEM, Oberkochen, Germany) equipped with a home-built heating stage. Nano-AES experiments were carried out using an Oxford/Omicron Nano-Auger system furnished with an ultra-high vacuum chamber (base pressure ~ 10–10 Torr), a field emission source, and a multi-channel hemisphere energy detector, as well as a heating specimen holder. The electron beam which can be focused to ~10 nm diameter enabled probing chemical composition in the nanoscale. In-situ analyses were conducted during the ramping up of the temperature from room temperature to 130 °C (~20–50 °C/min) and maintaining at the temperature for 1 h, under vacuum (<10^−6^ Torr).

## 3. Results and Discussion

**Figure 1:**
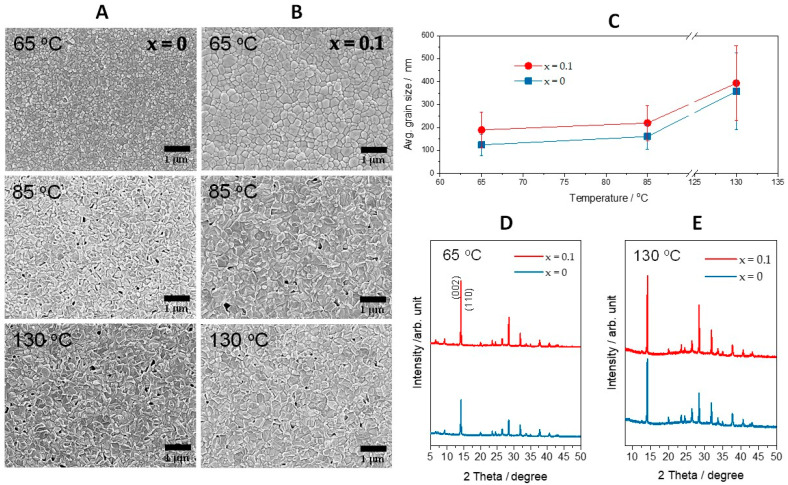
Corresponding In-situ SEM images of MAPbI_3−x_Cl_x_ films imaged at various growth temperatures, (**A**) x = 0 and (**B**) x = 0.10, respectively. (**C**) Approximate average grain size during annealing process from in-situ SEM images. X-ray diffraction results of MAPbI_3−x_Cl_x_ (x = 0, 0.10) from (**D**) soft baking at 65 °C for 1 min and (**E**) hard baking at 130 °C for 5 min.

Perovskite layers with a composition of MAPbI_3−x_Cl_x_ were prepared on TiO_2_ layers to make similar conditions for the grain growth of perovskite film that are used for optoelectronic devices, as the surface state of the substrates significantly affects the grain growth behavior. To observe the diffusion or sublimation of chloride ions, SEM analysis was performed in-situ while increasing the baking temperature. Figure 1A,B shows the in-situ SEM images of MAPbI_3−x_Cl_x_ (where x = 0, 0.10) samples recorded at various growth temperatures. The soft baking was performed at 65 °C to remove the solvents or anti-solvents and nucleate the perovskite crystallites. As shown in Figure 1B, at 65 °C MAPbI_3−x_Cl_x_ samples (where x = 0.1) displayed a larger grain size compared to the chlorine-free sample. This is not the difference in grain size during the hard-backing process for grain growth, but the difference in grain size during the soft-baking process. Additionally, soft baking was also performed at a 45 °C, and the result was the same as soft baking at 65 °C, suggesting chloride-induced grain growth. During the hard baking of MAPbI_3−x_Cl_x_ samples at a higher temperature, precipitates began to appear (85 °C under Figure 1A,B). In the case of x = 0 composition, precipitation started at about 90 °C, but in the case of x = 0.1 the composition precipitation started at about 80 °C. This means that the chlorine-related compound was volatilized at lower temperature. When increasing baking temperature above precipitation temperature (130 °C under Figure 1A,B), regardless of composition (x = 0, 0.1) the grains started to converge as a continuous network with the generation of pin holes. As shown in Figure 1C, the grain size difference was larger at the lower temperature, and the difference in grain size was lower at the higher temperature, asserting chlorine-induced grain growth even at lower temperatures. This phenomenon can be explained by the difference in phase formation energy (E_f_). When the E_f_ is low, a phase is formed at a lower temperature and the remaining energy can be used for grain growth. The phase formation energy can also be explained by the difference of the phase formation enthalpy (H_f_). The H_f_ of MAPbI_3_ is 34.50 ± 1.10 kJ/mol, and the H_f_ of MAPbCl_3_ is −9.03 ± 1.68 kJ/mol [23,24]. According to the first law of thermodynamics, ΔG = ΔH − TΔS, where TΔS is negligible in the experimental conditions, so ΔG ≈ ΔH. A negative Gibbs-free energy value means a spontaneous reaction and a stable state. Thus, the MAPbCl_3_ phase is more stable thermodynamically. Therefore, it can be deduced that the more chlorine is added to MAPbI_3_, the more thermodynamically stable it is and the E_f_ becomes lower.

The crystallization behavior and phase formation of MAPbI_3−x_Cl_x_ thin films was evaluated by recording XRD patterns. Figure 1D shows the XRD data of MAPbI_3−x_Cl_x_ (x = 0, 0.1) samples soft baked at 65 °C. After soft baking, it was confirmed that the MAPbI_3_ phase was formed in all the compositions regardless of the chlorine amount. A closer observation reveals that the (002) and (110) planes in MAPbI_3−x_Cl_x_ with x = 0.1 composition were larger than pure MAPbI_3_. This is in good agreement with the grain growth observed in SEM analyses (65 °C under Figure 1A,B). Besides, intermediate phase diffraction peaks of PbI_2_-DMSO and PbCl_2_-DMSO were seen at lower 2θ angles < 10° [25]. The MAPbI_3_ phase was formed because chlorine was added in an amount smaller than the solubility limit. The calculated and experimental solid solubility limit of chlorine in the MA site of MAPbI_3_ is about 4 at% [26,27]. Therefore, chlorine can be present, as the composition x = 0.10 is about 3.3 at%. Figure 1E shows the XRD data of a sample obtained by hard baking MAPbI_3−x_Cl_x_ (x = 0, 0.1) thin films at 130 °C. After hard baking at 130°C, the diffractions from the intermediate (PbI_2_/PbCl_2_-DMSO) phase disappeared in both of the MAPbI_3−x_Cl_x_ (x = 0, 0.1) compositions and a no-chlorine-related phase was observed in both the samples, consistent with the reported literature [28]. Interestingly, intensities of all diffraction peaks of x = 0.1 composition were higher than those of the chlorine-free composition (x = 0), suggesting larger grain growth, as illustrated in the SEM analysis (Figure 1A,B).

**Figure 2:**
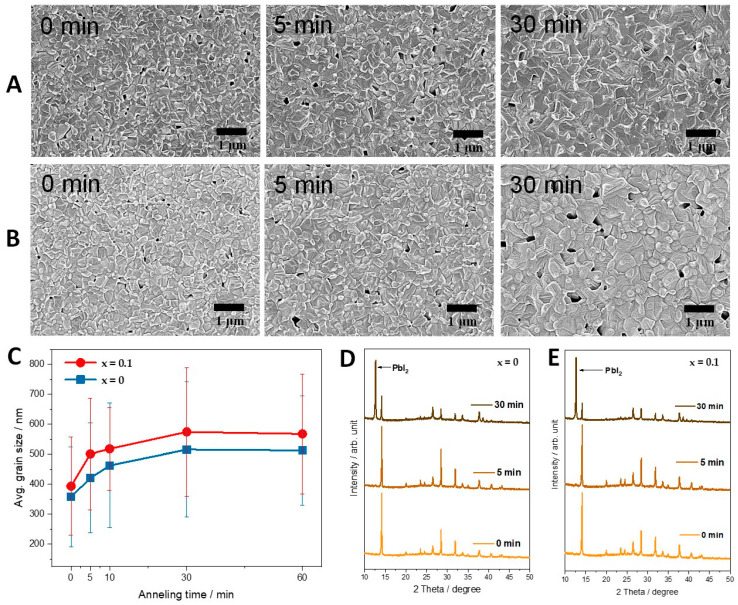
In-situ SEM images obtained by annealing of MAPbI_3−x_Cl_x_ films at 130 °C over a period of time. (**A**) x = 0, (**B**) x = 0.10, and (**C**) corresponding grain size distribution when samples reached and sustained 130 °C. X-ray diffraction results of MAPbI_3−x_Cl_x_ (x = 0, 0.1) annealed at 130 °C with increasing annealing time (**D**) x = 0, (**E**) x = 0.10, respectively.

To observe the volatilization behavior of chloride ions, SEM analysis was performed in-situ while increasing the baking time at a fixed temperature of 130 °C. Figure 2A,B shows the in-situ SEM images of MAPbI_3−x_Cl_x_ (x = 0, 0.1) thin films annealed at 130 °C for various (0, 5, and 30 min) time intervals. The top-view surface microstructure was observed to be same irrespective of the chlorine amount. However, it was seen that long-term annealing resulted in pin-hole formation in the x = 0.10 composition at a faster rate than x = 0, owing to chloride ion sublimation. Figure 2C shows the grain size of MAPbI_3−x_Cl_x_ (x = 0, 0.1) thin films by in-situ annealing. After 5 min of annealing at 130 °C, the grain size of MAPbI_3−x_Cl_x_ (x = 0.1) was comparatively larger than MAPbI_3−x_Cl_x_ (x = 0). This is because at a higher temperature the diffusion of chloride ions is much faster than iodide ions, resulting in more space for larger grain growth. Indeed, long-term baking converges large grains into a long network with the formation of precipitates near the grain boundaries. The evaporation of chloride ions and the presence of precipitates can be understood by XRD data in Figure 2D,E. Besides, after long-time annealing (30 min) at 130 °C, XRD results of MAPbI_3−x_Cl_x_ (x = 0, 0.1) thin films show PbI_2_ formation. In the case of x = 0.1 composition, PbI_2_ formation was observed at a shorter time than the chlorine-free composition (5 min data under Figure 2E), and after 30 min of baking the signature peak (002) intensity of x = 0.1 was lower than x = 0. The precipitation of PbI_2_ at a lower annealing time in x = 0.1 means that methylammonium (MA) was deficient and the chlorine was volatilized with MA, i.e., the MACl gas phase [29]. Hence, PbI_2_ was precipitated through grain boundaries, implying that the chlorine-related phase could be expected to evaporate through grain boundaries. To confirm this, n-AES analysis was performed in-situ to determine the difference in chlorine concentrations at the grain interior and grain boundary.

**Figure 3:**
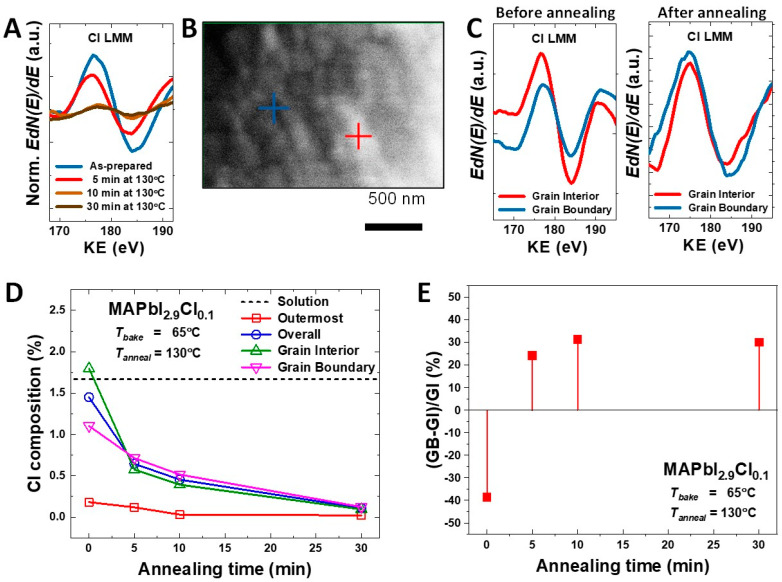
Nano-auger electron spectroscopy (n-AES) from MAPbI_3−x_Cl_x_ (x = 0.10). (**A**) Auger peaks of chlorine from outermost surface during the annealing process, (**B**) in-situ SEM imaging under the n-AES mode. Cross marks point out the regions where the auger signals were collected: the blue cross is for grain boundary and the red cross is for grain interior. (**C**) Auger peaks collected from grain interior (GI) and grain boundary (GB) before/after annealing at 130 °C for 10 min, (**D**) amount of chlorine at various points as the function of annealing time, (**E**) relative amount of chlorine detected from GB and GI ((GB − GI)/GI × 100%).

Figure 3A displays the quantitative change in the chlorine amount of representative MAPbI_3−x_Cl_x_ (x = 0.1) samples at the outermost surface with baking time given by conventional AES measurements. When baking was performed at 130 °C, we found that a large amount of chlorine had volatilized following 30 min of baking time. Then, a few atomic layers were removed from the outermost surface by gentle Ar ion milling to clearly observe the compositional difference of the grain interior and grain boundary. The reduced chlorine is attributed to evaporation of MACl, as the evaporation temperature of is about 100 °C [29,30]. Figure 3B shows a typical in-situ high-resolution SEM image under the n-AES mode where the e-beam current is much higher than that for standard SEM imaging, with the marks pointing to the regions where Auger signals were collected under the focused beam. Figure 3C illustrates the quantitative difference in the amount of chlorine between grain interior and grain boundary of MAPbI_3−x_Cl_x_ (x = 0.1) samples before/after annealing at 130 °C for 10 min. Before annealing, the as-formed MAPbI_3−x_Cl_x_ (x = 0.1) film exhibited higher concentration of chloride ions in the grain interior than grain boundaries. After gentle annealing at 130 °C for 10 min, grain interior chloride ions diffused out to grain boundaries via chlorine-to-iodine switching. This is likely because the ionic radius of chlorine is lower than the iodine, which permits intra-grain ions interdiffusion. Figure 3D shows the average chlorine percentage of MAPbI_3−x_Cl_x_ (x = 0.1) film in the solution, outermost surface, grain interior, and at the grain boundaries as a function of annealing time. The overall chlorine percentage in the as-grown MAPbI_3−x_Cl_x_ (x = 0.1) film was 1.5%, which is similar to the precursor solution (1.67%). The overall chlorine content was gradually decreased to 0.15% when annealed up to 30 min. This clearly implies that most of chloride ions were removed from MAPbI_3−x_Cl_x_ (x = 0.1) by evaporation in the form of MACl gas. Additionally, the chloride ions at the grain interior and grain boundaries also gradually decreased with longer annealing time. Although overall chlorine content was volatilized with an increase in annealing time, the chlorine content at the grain interior and grain boundaries was reversed after 5 min, as shown in Figure 3E. The ratio of chlorine composition difference at the grain interior and grain boundaries was less than 30% and it remained constant until 10 min of annealing time. This implies that the chloride-ion diffusion at a lower annealing time plays an important role in the grain growth of MAPbI_3−x_Cl_x_ (x = 0.1) thin films, which is in good agreement with XRD and SEM grain growth analyses (Figure 2). Importantly, a small fraction of chloride ions still remained at grain boundaries even after the annealing process of 30 min. This small fraction of inherent chlorine content is expected to be one of reasons for large grain growth and improved diffusion coefficient of mixed halide MAPbI_3−x_Cl_x_ compared to pure MAPbI_3_ composition. In support of previous reports [31,32], the in-situ experiment results presented here further suggest that the presence of chlorine precursors in MAPbI_3_ thin film formation may play an important role in grain growth, which is closely relevant to engineering morphology and electrical properties that are desirable for photovoltaic devices.

## 4. Conclusions

In summary, the incorporation of a small amount of chlorine increases grain size of resultant MAPbI_3−x_Cl_x_ thin films compared to pure MAPbI_3_. The mixed halide MAPbI_3−x_Cl_x_ phase is thermodynamically more stable and occurs at a lower formation energy than MAPbI_3_. Microstructure analyses by in-situ n-AES suggest that chloride ions inside the grain interior diffuse out to grain boundaries prompting faster grain growth of MAPbI_3−x_Cl_x_ thin films. At a lower baking temperature, the chlorine gradient is high at the grain interior, and when the MAPbI_3−x_Cl_x_ phase is baked for a long time, it changes to an MAPbI_3_-like microstructure due to the volatilization of chloride ions. Although the XRD results could not prove the chloride content in the MAPbI_3−x_Cl_x_ phase, the in-situ n-AES study concluded that a small fraction of chlorine was still present even after the longer baking time.

## Data Availability

The data presented in this study are available on request from the corresponding author. The data are not publicly available due to privacy restrictions.
